# Applications of drug delivery systems, organic, and inorganic nanomaterials in wound healing

**DOI:** 10.1186/s11671-023-03880-y

**Published:** 2023-08-22

**Authors:** Samantha Lo, Ebrahim Mahmoudi, Mh Busra Fauzi

**Affiliations:** 1 Centre for Tissue Engineering and Regenerative Medicine, The National University of Malaysia/Universiti Kebangsaan Malaysia, Kuala Lumpur, Malaysia; 2 Faculty of Engineering and Built Environment, The National University of Malaysia/Universiti Kebangsaan Malaysia, Selangor, Malaysia

**Keywords:** Nanomaterials, Wound healing, Drug delivery systems, Organic nanomaterials, Inorganic nanomaterials

## Abstract

The skin is known to be the largest organ in the human body, while also being exposed to environmental elements. This indicates that skin is highly susceptible to physical infliction, as well as damage resulting from medical conditions such as obesity and diabetes. The wound management costs in hospitals and clinics are expected to rise globally over the coming years, which provides pressure for more wound healing aids readily available in the market. Recently, nanomaterials have been gaining traction for their potential applications in various fields, including wound healing. Here, we discuss various inorganic nanoparticles such as silver, titanium dioxide, copper oxide, cerium oxide, MXenes, PLGA, PEG, and silica nanoparticles with their respective roles in improving wound healing progression. In addition, organic nanomaterials for wound healing such as collagen, chitosan, curcumin, dendrimers, graphene and its derivative graphene oxide were also further discussed. Various forms of nanoparticle drug delivery systems like nanohydrogels, nanoliposomes, nanofilms, and nanoemulsions were discussed in their function to deliver therapeutic agents to wound sites in a controlled manner.

## Introduction

In this day of age, skin wound healing has been an ever-pressing medical topic on a global scale in the pursuit of an effective treatment for it. Skin wounds do not only come from physical damage infliction, but can be a resulting complication from pre-existing medical conditions such as diabetes, obesity, and genetic disorders, which may also impair wound healing [1]. Data have shown that in various European countries, the percentage of obese people has increased from 10 to 40% over the past 10 years [2]. It is estimated that in developed countries, roughly 5% of the population are affected by skin wounds, of which 40% experience complications in wound healing [3]. In the United States of America (USA), type 1 and type 2 diabetes is expected to rise up to 30% by 2030 [4]. The average additional cost for wound treatment per patient after comorbidities is USD 3500. Around 15% of Medicare users in the USA have a wound or wound infection, which results in costs of up to USD 11,800 on average per patient [5]. Impaired wound healing as a result of comorbidities is also expected to rise within the coming years, which will increase the prevalence of acute and chronic wounds [1]. These skin wounds do not only impact the patient’s physical health which includes an increased risk of infection and pain, but also affect their mental health causing low self-esteem, emotional stress, and depression [6]. While the common wound dressing such as gauze and cotton aids in ensuring the skin wound area is dry and acts as a barrier to contaminants, dehydration as a result of excess moisture absorption can cause pain to the patients due to skin adhesion, in addition to providing only passive wound healing [7].

There are various modern wound dressings currently available in the market in various forms, such as films, foams, and hydrocolloids. Film dressings like Tegaderm™, Opsite™, and Bioclusive™ are very elastic and flexible, adapting to any shape without the need for taping [8]. Film dressings are more suitable for surface wounds with minimal exudate since they frequently have a restricted ability to absorb. Lyofoam™ and Tielle™ foam dressings are good options since they can absorb a lot of exudate, making them appropriate for wounds such as lower leg ulcers. However, foam dressings require frequent replacement and are inappropriate for dry wounds with little to no exudate. Hydrocolloid dressings like Comfeel™, Granuflex™, and Tegasorb™ offer exudate absorption and wound debridement capabilities. Hydrocolloid dressings are also easily permeable to water vapour and protect against bacterial infections. Conversely, this form of dressing might not be appropriate for wounds with a lot of exudates and neuropathic ulcers; therefore, it is frequently utilised as a secondary wound dressing instead [9].

Biomedical wound dressings are highly researched and sought after, which demonstrate various advantages in comparison to traditional wound dressings such as effective moisture absorption, maintaining an adequate amount of exudate and light adhesion on the wound site, promoting active wound healing with the incorporation of bioactive compounds as well as prevent infections [10]. Due to the various complications that come with skin wounds, the medical field is constantly researching new methods and materials in an effort to develop effective treatments, one of which includes nanomaterials for wound healing.

Nanomaterials have been present in nature since the beginning of the planet. They are defined as materials of organic, inorganic, and mixed sources presenting unique physical and chemical properties from their nanometer size. Natural nanomaterials are classified as nanomaterials made in nature without the interference of human activity [11]. However, naturally derived nanomaterials can also be created through human intervention from natural resources, which involves converting natural materials into nanometer sizes. In recent years, nanomaterials usage in various fields such as medicine and engineering has been on the rise due to better performances in comparison to regular sized materials. However, many nanomaterials used in these applications are from non-biodegradable sources, which may contribute to environmental pollution [12]. Hence, using natural or naturally sourced nanomaterials provides an eco-friendly method of obtaining nanomaterials and may end the production pollution cycle, while still providing superior benefits in various applications [13].

Nanomaterials can be a favourable choice in healing wounds due to its high surface area to volume ratio from its nano size, which serves for better drug encapsulation as well as its regulated control release. Nanomaterials may also have a better chance of contact with its supposed target such as receptors and intracellular components, thus providing more efficient and effective wound healing. Nanomaterial physicochemical aspects such as size, surface charge and hydrophobicity are also modifiable based on specific targets for optimal wound healing. For effective wound healing, the nanomaterials chosen should innately contain tissue repair properties and can act as drug delivery systems to carry therapeutic components [14]. Korrapati and colleagues also stated that ideal nanomaterials should be easily designed and modified, with a preference for naturally-source materials, possess biodegradability and biocompatibility properties, as well as compatibility with physiological solutions chemically [15].

## Physiology of wound healing

**Fig. 1:**
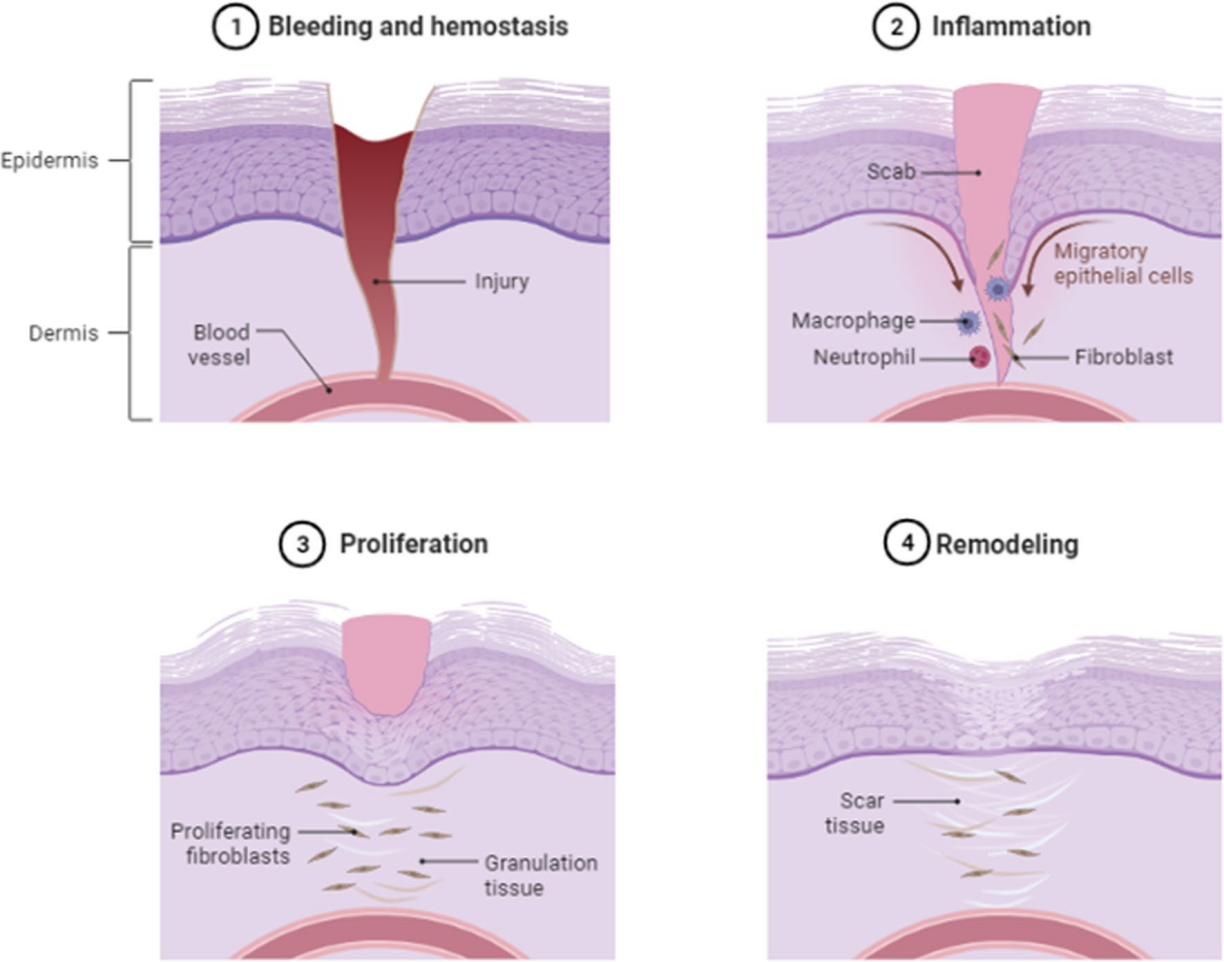
Summarised wound healing phases. Adapted from “Wound Healing”, by BioRender.com (2023). Retrieved from https://app.biorender.com/biorender-templates

Wound healing often refers to skin damage, to which various factors aid in its repair. Skin is the largest organ in the human body, designed to protect against a variety of environmental factors such as infections, superficial damage, ultraviolet radiation, and temperature changes. However, skin wounds are inevitable throughout the course of living, hence having an optimal repair mechanism is crucial [16]. When a skin wound occurs, the normal healing stages involve haemostasis, inflammation, proliferation, and remodelling (Fig. 1). In this case, nanomaterials can be applied to all phases of wound healing, accelerating its progression, preventing infections, and improving scar formations.

### Haemostasis

When a wound is inflicted, haemostasis occurs, the wound constricts to prevent blood loss by narrowing the damaged blood vessels through the increase of cytoplasmic calcium levels in smooth muscle cells present in the vessel wall, inducing contraction. This leads the reduced oxygen transfer to tissues, inducing hypoxia and acidosis which further triggers the release of vasoactive metabolites for vasodilation and blood vessel smooth muscle relaxation. The vasodilation together with vascular permeability from mast cell histamine release encourages the next phase, the inflammatory phase, to take place by allowing the influx of immune cell entry [17]. Intrinsic pathways also facilitate the release of activated platelets for fibrin clot formation. The haemostasis phase occurs immediately upon wound infliction and functions to restore homeostasis through re-establishing vascular structure, forming a protective barrier against the external environment and prevent excessive blood loss. Imbalances in this meticulous process threaten mortality from thrombotic or haemorrhagic conditions [18].

However, the addition of nanomaterials at this stage of wound healing may prove beneficial. Nanomaterials contain haemostatic qualities, such as nano sponges’ porous formation aid in exudate absorption. Nanomaterials can protect the surface of the skin wound and accelerate blood clotting. Nanomaterials also contain electrostatic adsorption properties, which encourage the recruitment of various blood cells such as platelets, erythrocytes, and leukocytes, as well as the adhesion of fibroblasts together with fibrin crosslinking at the wound site [19, 20]. For instance, Saikia and colleagues studied the effects of silica nanoparticles, which are inorganic nanoparticles, and its influences on platelet adhesion under flow conditions. They observed cellular surface interactions of these nanoparticles with the upregulation of platelet endothelial cell adhesion molecule 1 (PECAM-1) [21]. Different nanomaterials can also benefit haemostasis with different mechanisms of action. Carbon nanotubes, another inorganic biomaterial, improve wound healing by inducing platelet activation and aggregation, as well as upregulating thrombosis [22]. Nanomaterials applied at the haemostatic phase of wound healing act as an active wound dressing by accelerating through the wound healing phases, which are largely more beneficial than traditional dressings such as gauze and tulle, requiring frequent changes to avoid healthy tissue maceration [23].

### Inflammation

In the inflammatory phase, the immune system plays a role in preventing infections of the wound. Within an hour upon wound infliction, neutrophils are recruited and act as the first line of defence from pathogens, instigated through chemical signalling. Neutrophils function through phagocytosis and degranulation against pathogens. Neutrophils undergo phagocytosis by engulfing and digesting the invading pathogen. The process of degranulation occurs as neutrophils release toxic chemicals such as lactoferrin and proteases to further eliminate pathogens. After neutrophils have served their functions, they will be removed through apoptosis or macrophage phagocytosis. After 48–72 h of wound infliction, macrophages are recruited through chemical signals released from the wound site. Macrophages are an important aspect of wound healing as it contains various growth factors needed for wound repair. Macrophages contain growth factors such as transforming growth factor- beta (TGF-$\upbeta$) and epidermal growth factor (EGF) which aids in increasing re-epithelialisation, collagen formation, and angiogenesis [24]. Following macrophages, lymphocytes are then recruited to the wound site, managing the continuation of the wound healing process through releasing extracellular matrix (ECM) components and collagen deposition to aid skin reconstruction. Lymphocytes also aid in the reduction of immediate scar formation during skin wound healing [25]. The wound healing process will remain in the inflammatory phase as needed to remove pathogens present in the wound. However, wound sites prolonged in the inflammatory phase may lead to improper wound healing progression and severe scar formation.

In this phase, the application of nanomaterials on the wound site may be beneficial, especially for chronic wounds which often remain in the inflammatory stage. Chronic wounds such as diabetic ulcers remain in a longer healing duration due to hyperglycaemia, causing difficulties in angiogenesis as well as reduced oxygen and nutrients to the site of injury. Chronic wounds are in constant inflammation, prone to bacterial infections, and have downregulated growth factors. Because of this, various bioactive and non-bioactive components can be included into healing wounds at the inflammatory stage [26]. Bioactive molecules like growth factors, proteins, mesenchymal stem cells, and drugs can be incorporated into nanomaterials. Zhang et al. demonstrated electrospun dimethyloxalylglycine (DMOG)-embedded poly($\upepsilon$-caprolactone) (PCL) fiber (PCLF/DMOG) meshes applied onto diabetic rat wounds and found to have downregulated pro-inflammatory factors as well as upregulated anti-inflammatory factors and growth factors [27]. Comparatively, non-bioactive elements such as metal ions, chitosan nanoparticles, and silica nanoparticles often offer antibacterial and antioxidant properties. One example includes Ahmed R. and colleagues fabricated electrospun chitosan/polyvinyl alcohol (PVA)/zinc oxide nanofibrous mat for diabetic wounds. The biomaterial contains antibacterial activity against *E. coli*, *P. aeruginosa*, *B. subtilis*, and *S. aureus*, as well as exhibited antioxidant potentials and upregulated wound healing process [28].

### Proliferation

The next wound healing phase after haemostasis and inflammation is the proliferative phase. Proliferation in wound healing involves the process of skin reparation, which consists of angiogenesis, granulation tissue formation, re-epithelialisation, and wound constriction [29].

#### Angiogenesis

Angiogenesis involves the reformation of the vascular network and is a significant process for the delivery of oxygen and nutrients to wound healing. The wound site initially does not contain any vascular networks, solely relying on neighbouring healthy capillaries for diffusion. The process of angiogenesis begins after the formation of a haemostatic plug, with the release of vascular endothelial growth factor (VEGF), platelet-derived growth factor (PDGF), and fibroblast growth factor (FGF) from platelets. VEGF is released due to hypoxic wound site conditions, aiding in endothelial cell recruitment for neovascularisation and blood vessel repair [30]. Mixed metalloproteinases (MMPs) also play a role in wound healing, which are enzymes induced through the presence of hypoxic-conditioned neutrophil recruitment, encouraging angiogenesis through releasing VEGF and ECM reconstruction [31]. New blood vessels are formed from this process, which permeates the wound site originating from neighbouring blood vessels.

#### Granulation tissue formation

After wound infliction, growth factors such as TGF-$\upbeta$ and PDGF from the haemostatic plug enhances the recruitment of fibroblast to the wound site. After 3 days, the fibroblast-rich wound will have an abundance of ECM proteins, aiding in collagen assembly and fibronectin. This results in a fibrous tissue with an extensive vascular network known as granulation tissue, which substitutes the previous clot formed. After the proper establishment of the ECM, fibroblasts are converted to myofibroblasts, with pseudopodia functioning to assist wound contraction through connecting with collagen and fibronectin present in the microenvironment. Myofibroblasts are also involved in angiogenesis through MMPs activity regulation [17, 32].

#### Re-epithelialisation

Epithelial cells respond quickly to wound inflictions, as upon injury the bordering cells differentiate to cover the wound and connect with the matrix formed underneath. The process of re-epithelialisation is important as it acts as the defining step of a healed wound by covering the since-removed epithelial skin layer. Epithelial cells gain kinesis through epithelial-mesenchymal transition (EMT), allowing for motion in wound closure [33]. Cytokines are also present in varying concentrations during re-epithelialisation, aiding in epithelial cell morphology alteration, changing from cells with motility to a proliferative stage for repopulation at the wound site, thus completing the wound healing process [34]. This process is direct for primary intention wound closure. For a secondary intention wound, however, significant wound contraction must occur at the beginning before the re-epithelialisation process can begin. If the wound site is too large and wound contraction cannot occur naturally, skin grafts are available to aid the process [17].

#### Wound constriction

After re-epithelialisation is complete, myofibroblasts begin to aid in wound retraction after 1 week of wound closure. Actin and myosin proteins play a role in bringing cell bodies closer to reduce the distance needed to overcome during healing. Contraction rates average 0.75mm per day depending on wound morphology such as wound size and shape. Distortions on the skin known as contractures can occur if wound retraction does not progress normally [17, 35].

### Remodelling

Remodelling of the wound is considered the final process in wound healing, beginning at 2 weeks upon wound infliction and taking up to 2 years to complete. The purpose of the remodelling phase is to achieve a synthesis and degradation equilibrium, gaining maximum tensile strength. This phase primarily focuses on the organisation of all proteins and cellular components present to achieve normal skin structure. Granulation tissue is also remodelled into scar tissue, with type I collagen being converted into type III collagen, achieving the structural morphology of a scar. However, healed wounded skin will regain equal tensile strength as compared to normal unwounded skin, achieving a maximum of 80% while averaging at only 50% [36].

As we can see from the above detailing, the proliferative phase is a significant event in wound healing due to the recruitment of fibroblast for proliferation, blood vessel formation, and wound closure [37]. As with the previous phases, nanomaterials can also be applied to this phase. For instance, Losi et al. combined VEGF and basic fibroblast growth factor (bFGF) into poly(lactic-co-glycolic acid) (PLGA) nanoparticles, which successfully upregulated granulation tissue formation, collagen deposition, and complete re-epithelialisation [38]. With the myriad of nanomaterial varieties available for wound healing applications, different nanomaterials can be used to yield similar results. Chitosan-based copper nanocomposites applied on adult Wistar rat excised wounds demonstrated successful fibroblast proliferation, re-epithelialisation, and collagen deposition, which accelerated healing time [39].

Besides the reliance on wound healing physiology, various internal and external factors also contribute to its success or failure. External factors would include nutrition, smoking, infections, and wound management. Whereas internal factors such as age, chronic diseases, immunosuppression, and genetics all play a role in determining the rate and morphological aesthetic of healing wounds [17].

## Nanomaterial fabrication methods

**Fig. 2:**
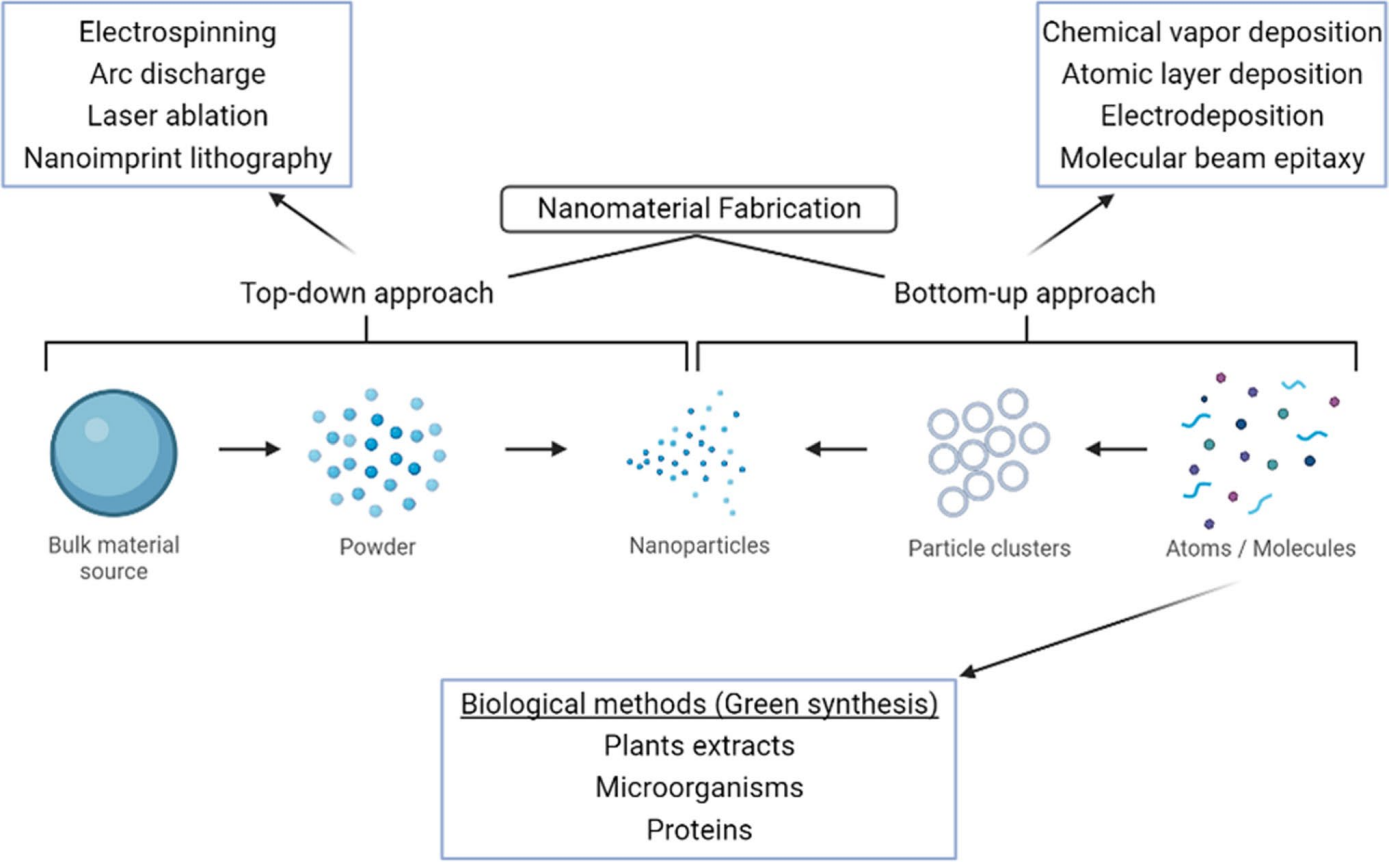
Summary of nanomaterial fabrication methods. Created with BioRender.com

The fabrication of nanomaterials typically follows two approaches: the top-down approach or bottom-up approach (Fig. 2). The top-down approach is a process that involves the breakdown of a larger polymer or material into smaller structures. This method includes processes such as electrospinning, arc discharge, laser ablation, and nanoimprint lithography. On the flip side, the bottom-up approach utilises smaller atoms or molecules assembled to form nanoparticles, involving processes like chemical vapor deposition, atomic layer deposition, electrodeposition, and molecular beam epitaxy [40]. While commonly utilised, these traditional fabrication methods pose serious consequences to our environment, as certain chemicals used and produced in these processes are toxic and not eco-friendly [41]. This invites the need for a different fabrication method, which is the green synthesis of nanomaterials.

Green synthesis is the fabrication of nanomaterials from biological sources such as microorganisms, plants, fungi, proteins, whole cells, etc. [42]. Green synthesis is a cost-effective, clean, environmentally friendly, and sustainable nanoparticle fabrication method in comparison to the traditional methods. It is used to replace dangerous chemical usage and aims to reduce toxic byproducts to protect human health and the environment [41]. There are various systems for the green synthesis of nanoparticles, such as green synthesis from enzymes and vitamins, microwave-assisted synthesis, and bio-based methods [43]. The following nanoparticles discussed below primarily involve green synthesis in their fabrication.

## Inorganic nanoparticles

Inorganic nanoparticles are defined as nanomaterials that possess minimal to no carbon within their structure, which can be composed of metal or non-metal elements [44]. Inorganic nanomaterials can be an attractive form of materials to be used in wound healing as it is typically inert, stable, and may possess characteristics such as optical and magnetic properties [45]. Inorganic nanomaterials can be divided into three categories: mainly metallic, polymeric, and ceramic nanomaterials, which are discussed below in relation to wound healing.

### Metal nanoparticles

Metal and metallic nanoparticles could be found in the earth’s core. However, due to its finite nature, a myriad of researchers are tapping into biological sources such as various plants and microorganisms for metallic nanoparticles. Upon green synthesis of metallic nanoparticles, it can be incorporated into many fields for a variety of usages, for example in biotechnology, agriculture, food and textiles, as well as wastewater treatment [46]. Some examples of metallic nanoparticles are mentioned below with involvement in wound healing properties.

#### Silver nanoparticles (AgNPs)

Silver nanoparticles (AgNPs) belong to the category of nanomaterials that can be derived from natural sources and have long been known to aid in skin wound healing. Previously upon the discovery of antibiotics, AgNPs were put aside in favour of use of the former. However, due to the onslaught of antibiotic-resistant bacterial strains in recent years, AgNPs have since regained its significance in the medical and research field [47]. These nanoparticles display broad-spectrum antibacterial properties and are less likely for antibacterial resistance induction. AgNPs work by reacting with SH protein groups and bacterial inactivation. The silver ions act on separating respiratory electron transport from oxidative phosphorylation, either affecting respiratory chain enzyme action or disrupting proton and phosphate membrane permeation [48]. AgNPs also notably have antiviral, anti-inflammatory, and antifungal properties, making it a favourable component in wound healing. AgNPs can be found in nature through the reduction process of certain plant, fungi, and bacterial extracts, reducing Ag ions to AgNPs [49]. These nanoparticles, known as green-synthesised AgNPs, can also be developed from herbal extracts such as *Propolis* [50], *Phytophthora infestans* [51], and *Biophytum sensitivum* [52]. The inflammatory properties of AgNPs have mostly been investigated because of the significance of the inflammatory phase in wound healing. AgNP-containing wound dressings may speed up the healing process by reducing inflammation through regulating cytokine production [53]. One study conducted by Al-Shmgani et al. synthesised AgNPs from *Catharanthus roseus* leaf extract demonstrated regulated bacterial and fungal growth, enhanced wound closure, and reduced wound size in vivo mice. During treatment, AgNP-treated wounds showed no signs of microbial contamination, bleeding, or pus development while control wounds showed noticeable inflammation. From the fourth day and after, the AgNP-treated group experienced distinct wound closure and decreased wound size, which improved throughout the course of the remaining treatment days in comparison to the controls. At the final stage of the study, the AgNP-treated wound had closed to a degree of about 98%, compared to 85% for the control wound [54]. Another study by Wen et al. demonstrated the use of *O. chinensis* antibacterial endophytic fungus in synthesising AgNPs, successfully enhancing wound healing, display antibacterial properties and treating infected wounds on Sprague-Dawley rats. On day 14, the AgNPs group demonstrated a better overall state of the wound, with less scarring and alleviated redness indicating reduced inflammation. In comparison, the control group either had some scab remaining or had shed, however the wound was still inflammatory and scarred [55].

#### Titanium dioxide nanoparticles (TiNPs)

In leu of the topic on AgNPs usage, titanium dioxide nanoparticles (TiNPs) are also highly sought-after nanomaterials in focus on its antibacterial properties in wound healing. TiNPs differ from AgNPs in that these nanoparticles have excellent mechanical properties, has bactericidal effects on gram-positive and gram-negative bacteria, promote chemical activity due to its large surface area to volume ratio, resistant to corrosion as well as promotes cellular development [56]. To further elaborate on its antibacterial properties, the TiO_2_ particles function as photocatalysts by upregulating peroxidation of lipid membrane polyunsaturated phospholipids, hence reducing cellular respiratory activity leading to cell death within 20 min of induction. TiNPs have also been found to induce cellular damage more efficiently, hence providing better bactericidal activity [57]. Similarly to AgNPs, TiNPs can also be sourced from nature. Sankar et al. successfully synthesised TiNPs at room temperature with notable wound healing progression in Albino rats [58]. Another study conducted by Sivaranjani et al. synthesised TiNPs from *Moringa oleifera* leaf extract and applied to wounds in Albino rats. The nanoparticles effectively reduced wound size more significantly than the control group, indicating efficient wound healing progression [56]. A reflection of the TiNPs’ strong antibacterial potentials, as demonstrated by the findings of the in vivo and in vitro antibacterial assays, is the absence of irritation and/or discomfort at the wound site during treatment and the considerable acceleration of wound contraction and re-epithelialisation rates, primarily seen in the remodelling phase of wound healing [59].

#### Copper oxide nanoparticles (CuNPs)

Besides that, copper oxide nanoparticles (CuNPs) can be incorporated into wound healing dressings or aids due to its antibacterial activity, ability in upregulating transcription and growth factors as well as promote angiogenesis [60]. CuNPs antibacterial properties are credited to nanoparticle electrostatic binding to the phospholipids and polyanionic lipopolysaccharides on bacterial outer membrane, disrupting complement activation and evasion of bacteria [61]. CuNPs effectively prevent bacterial infection at wound site, hence improving tissue integrity restoration [62]. Borkow et al. studied the effects of CuNPs wound dressing on genetically engineered diabetic mice. They found that hypoxia-inducible factor (HIF)-1 alpha, epidermal growth factor receptor, platelet-derived growth factor, and interleukin VIII were just some of the proteins and receptors upregulated with the wound dressing, which aided in wound healing progression [60]. CuNPs can also be found in nature and extracted from various plants such as *Gloriosa superba* [63], *Malva sylvestris* [64], *Terminalia arjuna* [65], and *Calotropis gigantean* [66]. Naturally sourced CuNPs are important nanoparticles as it plays a role in biopharmaceutical industries as well as in reversing environmental pollution [67]. Another study by Andualem et al. demonstrated the synthesis of CuNPs from *Catha edulis* leaf extract together with a copper nitrate trihydrate precursor. The resulting nanoparticles displayed antimicrobial activity towards *S.aureus*, *S.pyogenes*, *E.coli*, and *K.pneumonia* [68]. The healing process of copper oxide-containing wound dressing on genetically modified diabetic mice was examined through the expression of 84 genes involved in angiogenesis, which is mainly observed in the proliferative phase of wound healing. At 5 and 10 days after copper oxide dressing application, the expression of Hif-1 alpha, which is a protein that is secreted under acute circumstances such as oxidative stress, increased in the mice, effectively increasing angiogenesis [69].

#### Cerium oxide nanoparticles (CeNPs)

Cerium is a rare earth metal that can exist in 3+ and 4+ states. In the nanoscale, cerium in both states and cerium oxide were combined on nanoparticle surfaces [70]. Cerium oxide nanoparticles (CeNPs) can also be green synthesised and sourced from various plants and microorganisms that are used in wound healing due to its antibacterial properties. CeNPs improve skin wound healing by promoting angiogenesis through the reduction of reactive oxygen species via mechanisms involving regulation of hypoxia-inducing factor 1$\upalpha$, improving the intracellular oxygen environment as well as upregulating VEGF [71, 72]. Pezzini and colleagues demonstrated the effects of CeNPs antioxidant properties on primary human skin fibroblasts from healthy subjects by studying the mitochondrial functionality in basal and oxidative conditions. The results showed no cellular toxicity and significant antioxidant activity, making CeNPs a promising wound healing material [73]. Various other studies were conducted with the incorporation of CeNPs into wound healing biomaterials, including a study by Kalantari et al., to which they incorporated CeNPs sourced from Zingiber officinale extract in chitosan/PVA hydrogels. The hydrogel composite displayed antibacterial properties with human dermal fibroblast viability for 5 days [74]. Besides wound healing, CeNPs can also be utilised in various other applications involving the need for other properties such as an anti-larvicidal, antimicrobial, anti-cancer, antioxidant, and photocatalyst [75].

With various metallic nanoparticles to choose from for the application of wound healing, there is a consensus that these nanoparticles largely contain antimicrobial properties, preventing skin wound infections and thus accelerating the wound healing process [76]. Hollow CeNPs with a rough surface and l-arginine incorporation are designed as an adjustable nanosystem for the haemostasis, inflammation, and proliferative stages. The coarse surface of these nanoparticles functions as a nanobridge to accelerate wound closure during haemostasis. The porous shell of the nanosystem simultaneously promotes the generation of reactive oxygen species, reducing wound infection and accelerating wound healing in the inflammatory stage. As a result of the nanosystem’s ability to mimic an enzyme, oxidative damage to the wound can be reduced, and the released l-arginine can be transformed into nitric oxide which supports the proliferation stage [77]. In terms of its effectivity, one study combined chitosan with zinc oxide, titanium dioxide, and silver nanoparticles, to which the researchers found that silver nanoparticles may have better antimicrobial properties. However, this statement was not proven consistently [78]. Further research is required to determine the metallic nanoparticles with the most efficient wound healing properties.

### Polymeric nanoparticles

#### MXenes

**Fig. 3:**
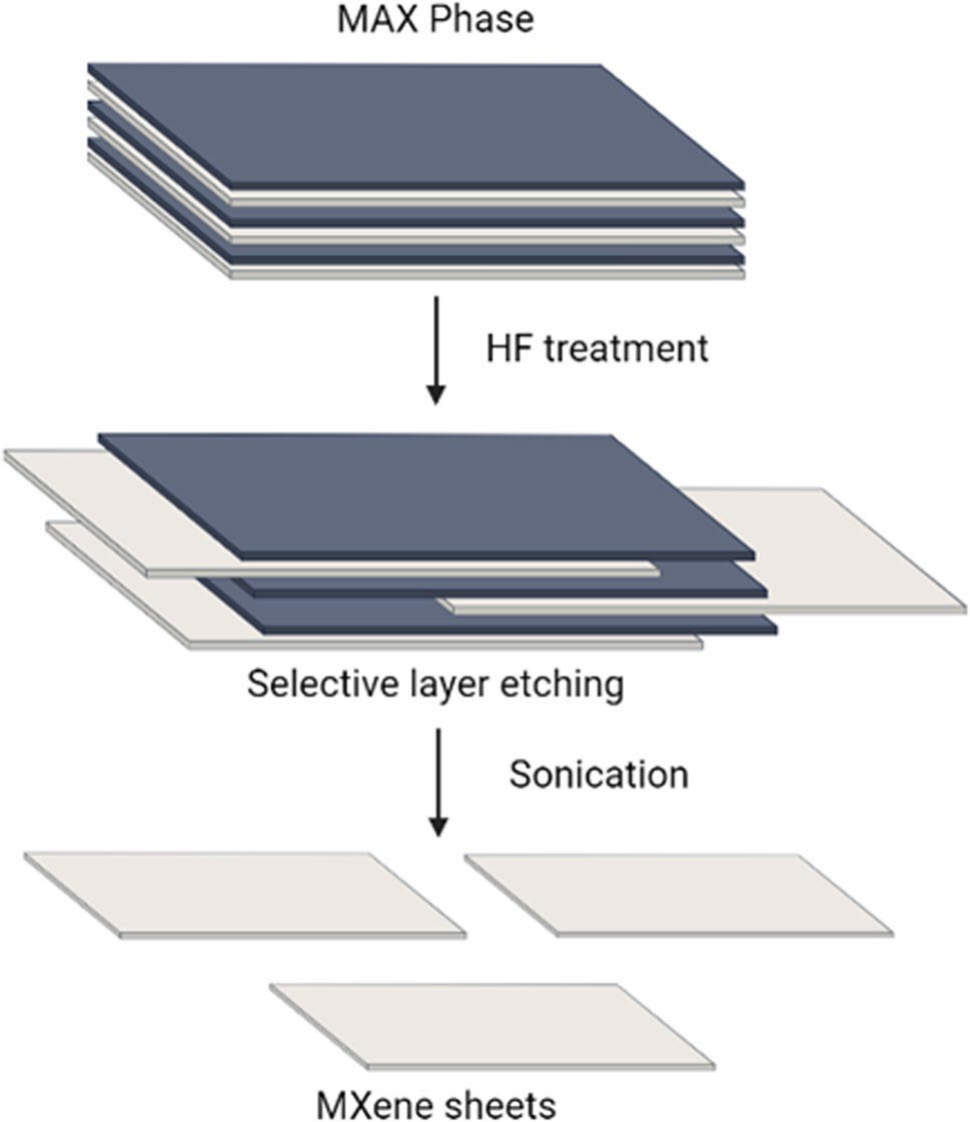
MXene sheet formation process. Created with BioRender.com

MXenes are a two-dimensional (2D) polymeric layered material composed of transition metal carbides, nitrides, and carbonitrides. The emergence of MXenes are largely due to the discovery of graphene and its contributions in material sciences, peaking interest in other forms of 2D materials. MXenes are typically formed from selective sp element layer etching from MAX phases, which are chemical delamination of 3D ternary or quaternary compounds (Fig. 3) [79, 80]. MXenes were discovered to be superior to graphene because it is naturally hydrophilic and has higher electrical conductivity than graphene produced through solution processing [81]. MXenes can be formed into various materials such as nanosheets [82]. MXenes exhibit properties such as high metallic conductivity, hydrophilicity, biocompatibility, large specific surface area, low diffusion barrier, and high ion transport capabilities [83]. MXenes are also antibacterial and have photothermal therapy and fluorescent imaging qualities, suited for biomedical applications [84, 85]. MXenes can be used in a myriad of applications, from batteries and supercapacitors to biosensors and cancer biomarkers [79]. The optical characteristics of MXenes, such as light absorption, emission, or scattering, have also received attention from researchers. MXenes’ potent light-harvesting ability makes them ideal for applications involving the conversion of light into heat, commonly known as phototherapy [86].

MXenes can also be used in wound healing applications, which will be the primary focus in this segment. L. Zhou and colleagues fabricated a 2D Ti_3_C_2_T_x_ MXene nanosheets that is antibacterial and multifunctional. They combined branched poly(glycerol-ethylenimine) (PGE) with Ti_3_C_2_T_x_ MXene@polydopamine nanosheets for antibacterial activity, conductivity, and skin-adhesive properties, together with oxidised hyaluronic acid for scaffold matrix support. As a result, the MXene scaffold displayed electrical conductivity, tissue-adhesion, antibacterial, haemostatic, and self-healing characteristics. It enhanced skin cell proliferation, presented anti-inflammatory properties, promoted angiogenesis, collagen deposition, granulation tissue formation, and vascular endothelial differentiation, all with minimal cytotoxicity [87]. Ti_3_C_2_T_x_ MXene nanosheets exhibit antibacterial activity via direct physical contact between the razor-sharp edges of the nanosheets and the membrane surfaces of bacteria, effectively damaging bacterial cells in three hours [88].

Another study by L. Jin et al. assembled temperature-responsive MXene nanobelt fibers (T-RMFs) with vitamin E for wound healing. The T-RMFs were fabricated using MXene nanosheets, polyacrylonitrile, polyvinylpyrrolidone composite nanobelts, and thermosensitive P(AAm-co-AN-CO-VIm) copolymer (PAAV)-coating layer. The MXene thermoresponsive qualities can be activated through near-infrared irradiation temperature control, causing the release of vitamin E through the polymeric coating layer interface relaxation. According to the results of the cell evaluation, during the culture phase, the cells on the T-RMFs showed significantly greater cell adhesion and proliferation compared to cells on the control fibres, displaying superior biocompatibility. The scaffold also displayed biocompatibility and high mass loading and surface area, demonstrating its excellent wound healing functions [89].

X. Xu and colleagues fabricated a MXene-based nanofibrous membrane with antibacterial properties for wound infection treatment. They electrospun a combination of the antibiotic amoxicillin (AMX), MXene, and PVA. The PVA construct functions to regulate AMX release, whereas MXene converts near-infrared irradiation to heat energy, hence upregulating AMX release to overcome wound site bacterial infections. The scaffold’s effective antibacterial activity has been confirmed through S. aureus in vitro testing and in vivo mouse models, as well as improving wound healing and providing a physical barrier against the environment. While remaining traceable after 48 h, the release of AMX gradually continues to be steady throughout time, suggesting that the PVA nanofibrous membrane was successful in controlling the release rate of AMX. The sustained drug release rate of AMX dramatically increases at 50$^{\circ }$C, mimicking the laser irradiation environment, and demonstrating that AMX discharge is temperature dependent. The continuous release of AMX contributes to the nanofibrous membrane’s long-term antibacterial action [90].

The effects of MXene-integrated microneedle dressings with adenosine encapsulation on wound repair were investigated. Utilising MXene’s photothermal conversion capability, the dispersion of preloaded adenosine can be increased by near infrared irradiation to preserve the activation signal close to the injury site. In the range of 0.001 mg/mL to 0.09 mg/mL, the absorbance value revealed a linear association with adenosine concentration. Adenosine and its associated receptors have been shown to promote fibrosis, matrix formation, and vascular development, which may aid in the healing of wounds. It was proven that the microneedle patches effectively increase angiogenesis, a process essential for the proliferative phase in wound healing, when used in vivo and in vitro [91].

#### Poly(lactic-co-glycolic acid) (PLGA)

One of the most commonly utilised biodegradable polymers in medical applications is poly(lactic-coglycolic acid) (PLGA), a copolymer of poly(lactic acid) and poly(glycolic acid). In many clinical applications, PLGA is the most appealing polymeric drug carrier due to its commercial availability, optimal degradation in physiological settings, the capacity to alter the physiochemical and surface characteristics, superior biocompatibility, and prolonged drug therapy release [92]. In wound care, PLGA itself can act as a wound healing agent as well as a drug delivery carrier. The lactic acid in PLGA could be broken down to lactate, to which was found that the use of exogenous lactate may expedite the angiogenesis and wound repair, to which PLGA was proposed to be the most beneficial polymer to use as a source of lactate. In comparison to the control group, there was a roughly 60% decrease at the injury site after 10 days [93].

In terms of drug delivery, PLGA is highly biocompatible with various other polymers and has the capacity to transport various kinds of therapeutic agents efficiently. A common dual-phase curve for pharmaceutical delivery is present in PLGA-based biomaterials. The rate of the initial burst phase is dependent upon the type of drug encapsulated, the drug concentration, and the hydrophobicity of the polymer [94]. The medication is gradually released from thicker layers of PLGA matrix during the second phase, during which water degrades the matrix. Chen et al. demonstrated bFGF encapsulation in PLGA and co-loaded onto polyvinylpyrrolidone microneedles with free ofloxacin. Due to the substrate’s rapid fragmentation, ofloxacin was released rapidly to prevent infection while the PLGA biomaterial remained in the wound. As PLGA degrades progressively, the encapsulated bFGF that was slowly released to aid in wound healing [95].

Another study demonstrated the combinatorial effects of PLGA nanoparticles, whereby layered-structure nanofibrous membranes of biodegradable PLGA were created using a combination of collagen, PLGA, and various antimicrobials, with PLGA/collagen serving as the superficial layers and PLGA/drugs serving as the core. The nanofibrous membranes were actively engaged in controlling bacterial infections and substantially sped up the initial phases of wound healing in a rat model of wound infection with *Staphylococcus aureus* and *Escherichia coli*. The treated group had greater wound healing, re-epithelialised epidermis with newly synthesised fibrous tissue plus newly synthesised connective tissue, and few inflammation-related cells in the epidermal layer and subcutis, according to histological analysis of the wound tissues [93].

#### Polyethylene glycol (PEG)

Polyethylene glycol (PEG) is a low-cost, water-soluble, linear polymer derived from the live anionic ring-opening polymerisation of ethylene oxide. Attaching PEG via covalent or noncovalent bonds can facilitate the distribution of proteins, peptides, and various other molecules without impairing their bioactivity. Along with PEGylation known as bioconjugation, PEG may be cross-linked to create porous hydrogels that can act as biocompatible matrices that resemble the ECM of tissues [96]. PEG and its copolymer hydrogels are useful scaffolding materials that have been utilised for tissue engineering as well as cell culture, regulated administration of drugs, and a number of other uses. PEG hydrogels are frequently used in medical devices, as bonding agents for wound closure, slow-release matrices for pharmaceuticals, wound healing, and act as a regenerative medicine medium [97].

One study fabricated a series of shear-thinning and self-healing poly(ethylene glycol)-block-polylactide nanoparticle hydrogels. Its diameter, Dh 80 nm, was small enough to promote gel formation by favouring polymer bridging between multiple nanoparticles rather than polymer wrapping around individual particles. Hydrogels with the longer PEG brush had better rheological characteristics and recovered from shear-thinning more quickly [98]. The formation of several types of nanoparticles by amphiphilic PEG-PLGA copolymers has also garnered attention. PEG-PLGA nanoparticles have a core architecture that is suited for encapsulating active ingredients with limited water solubility, as well as a surface hydrophilic corona that acts as a barrier contact between the centre and the external environment. PEG-PLGA NPs are thought to be viable drug delivery methods for either synthetic or natural medicines [99].

### Ceramic nanoparticles

#### Silica nanoparticles (SiNPs)

Ceramic nanomaterials are comprised of oxides, carbides, phosphates and/or carbonates with metalloids such as silicon [100]. Ceramic-based nanoparticles display properties such as a semi-crystalline nature, high surface area, large pore volume, and contain surface charge, all of which are suitable characteristics for the use of accelerating wound healing [101]. Silica nanoparticles (SiNPs) can be fabricated in four different methods: the sol–gel method, template-directed method, chemical etching, and microwave-assisted technique [102]. SiNPs also display a mesoporous structure, which is suitable for controlled drug delivery [103, 104]. While the mechanism of action of silicon is currently undetermined, it is hypothesised that it may be involved in the metabolism and/or structure of collagen formation and stability [105].

Pan and colleagues developed a mesoporous SiNP tissue adhesive for skin wounds and found that the biomaterial could upregulate the inflammatory response to accelerate wound healing and is subsequently removed upon tissue formation [106]. S. Quignard et al. utilised amorphous SiNPs in the controlled release of soluble silicic acid for wound healing. The study demonstrated the non-toxic property of silica, and also found that the positively-charged SiNPs encouraged fibroblast proliferation and migration and were internalised more effectively than silicic acid, indicating that SiNPs are efficient nanomaterials for intracellular delivery of bioactive compounds. While SiNPs mechanism of action on wound healing has not been confirmed, it is postulated to have metabolic and/or structural purposes in collagen synthesis and stabilisation [107].

SiNPs can act as a carrier for metal or metal oxide nanoparticles for antibiofilm properties against bacteria, in which SiNPs incorporated with copper dioxide and zinc dioxide presented photocatalytic activity against *E. coli* and *S. aureus* [108]. Mesoporous SiNPs layers behave as micro-reactors and can improve the antibacterial properties of AgNPs incorporated due to its uniform arrangement within the structure. It can allow a fast release of silver ions, increasing antibacterial performances against *E. coli* and *S. aureus* in comparison to silver nanoparticles alone [109]. Mesoporous SiNPs loaded with dimethyloxaloylglycine and aligned poly(lactic acid) nanofibers were experimented on chronic wound healing. In vivo investigation showed the composite scaffolds significantly accelerated the healing of diabetic wounds by enhancing angiogenesis, collagen deposition, and epithelialisation at the wound sites during the proliferative phase [110].

## Organic nanoparticles

### Collagen

Collagen is a widely studied biomaterial as it is a protein largely found in humans, comprising 20–30% of all proteins found in the body and provides cellular function regulation as well as structural support in the ECM [111, 112]. Collagen is an ideal material for wound healing as it is pro-haemostatic and proliferative for skin cells such as fibroblasts and keratinocytes, as well as provide biological cues for cell formation and behaviour [112, 113]. Collagen biomaterials also do not have biocompatibility issues, are biodegradable, non-toxic, and bioabsorbable.

Collagen is largely sourced from bovine, porcine, equine, or avian origins [114]. However, due to various potential complications such as bacterial infections, allergic reactions, and prion disease transmission, other collagen sources from marine life or bioengineered from plant or bacterial recombinant human collagen are considered [115]. While collagen can be fabricated into other forms such as sponges, sheets, tubes, powders, and foams, the current collagen nanofiber fabrication method is still primarily electrospinning [112]. Presently there are no green synthesis method for the production of nano collagen. However, collagen nanofibers are still an important naturally sourced biomaterial for wound healing, and its benefits should not be discounted for due to its fabrication method.

KS Rho and colleagues electrospun type I collagen in 1,1,1,3,3,3-hexafluoro-2-propanol, producing nanofibers at 460nm. The collagen nanofibers demonstrated excellent tensile strength. The biomaterial was tested on open wound healing in rats, and showed improvements in cell adhesion and early-stage wound healing rates [116]. Another study conducted by Rath et al. demonstrated electrospun collagen nanofibers with the incorporation of AgNPs for wound healing. They have successfully fabricated collagen nanofibers with diameter ranges between 300–700nm, together with the regulated release of silver ions. In vitro and in vivo studies revealed antimicrobial activity from the silver ions and that the combination of collagen nanofibers with AgNPs improved wound healing progress as compared to regular collagen nanofibers, together with upregulated re-epithelialisation, collagen deposition, and wound contraction [117]. The main mechanism of action of collagen biomaterials includes providing a porous scaffolding and fibrillar matrix for cell adhesion and migration [118].

### Chitosan

Chitosan is a natural polymer, specifically a polysaccharide macromolecule with cationic properties typically sourced from exoskeletons and crustaceans of arthropods. It is produced from chitin found in the aforementioned sources in a partial deacetylation process [7]. Its basic pH contributes to various ideal wound healing properties such as excellent biodegradation, biocompatibility, and relatively low cytotoxicity [119, 120]. Chitosan as a wound healing agent is also involved in the many stages of the process. It can promote neutrophil and macrophage migration to the wound site and possesses haemostatic qualities to the initial wound infliction [121]. Chitosan can also aid in reducing the appearance of scars and upregulate epithelial tissue regeneration [122].

From all the aforementioned wound healing properties, chitosan is an excellent choice for biomaterial fabrication. Chitosan as a wound healing biomaterial has a slower biodegradation rate compared to gelatin or collagen-based biomaterials. The rate can also be altered in accordance with application needs through fabrication with other polymers. Furthermore, chitosan-based biomaterials also present antimicrobial and antifungal properties [123]. Presently, like collagen, chitosan nanofibers are commonly achieved via electrospinning. However, due to chitosan’s high hydrogen bonding degree and crystallinity, this results in chitosan biomaterial having low water and organic solvent solubility.

To combat this, Qian et al. developed a green synthesis method to fabricate chitosan-based nanofibers by freeze-drying diluted chitosan aqueous solutions without utilising organic solvents or acidic solutions in high concentrations. The chitosan nanofibers produced diameter ranges of 100–700 nm, which can then be utilised for controlled drug release in wound healing [124]. AA Tayel and colleagues prepared nanochitosan from shrimp shell via mechanical pulverisation to create a composite together with fish collagen and henna extract. The results show that the composite exhibits antimicrobial activity towards skin pathogens by causing microbial lysis and deformation. The composite also upregulated wound healing progression in rats, making it suitable as a skin wound healing biomaterial [125]. Chitosan’s cationic character, which is caused by amino groups in N-acetyl glucosamine units, allows it to interact with positively charged lipids, phospholipids, polysaccharides, and proteins found in bacterial cell walls. Chitosan is suggested to exhibit antibacterial activity via chitosan amino groups engage electrostatically with the negatively charged cell membrane of the target, causing the cell wall to become permeable, leak, and apoptose [126].

In terms of wound healing, L929 and NIH3T3 mouse fibroblast cell lines were used to determine the effect of the chitosan-pectin-TiO2 dressing material on cell viability for a period of 3, 7, and 14 days. For NIH3T3 cells, cell viability at 3 and 7 days is greater than 97%; for L929 cells, it is 100% at 3 and 97% at 7 days. In vivo wounds treated with the chitosan-pectin-titanium dioxide nano dressing material exhibited enhanced levels of natural hyaluronic acid synthesis, fibroblast proliferation, and supported organised collagen deposition in the proliferative stage. In comparison to other groups and gauze dressing, the wounds treated with the chitosan-pectin-titanium dioxide dressing material healed more rapidly. For 3, 7, 11, and 16 days, the gauze-treated group’s wound closure rate is 17.45%, 46.98%, 82.87%, and 91.22%, respectively, while the chitosan-treated groups’ wound closure rates are 28.79%, 56.98%, 87.11%, and 94.98%, respectively [127].

Lin et al. created a drug-free, non-crosslinked chitosan/hyaluronic acid hybrid hydrogel to combine the many healing benefits of chitosan and hyaluronic acid of a diabetic wound infected with methicillin-resistant *Staphylococcus aureus* (MRSA). As a result, the composite hydrogel demonstrated exceptional reactive oxygen species elimination, boosted fibroblasts proliferation and migration, and had significant impacts on cell protection under oxidative stress. In diabetic mouse wounds with MRSA infection, the hydrogel greatly accelerated wound healing by eradicating MRSA infection and promoting angiogenesis, epidermal regeneration, and collagen synthesis [128].

### Curcumin

Curcumin is found in the rhizomes of *Curcuma longa* and is an age-old herbal medicine used for its antioxidant, anti-cancer, and anti-inflammatory properties. Curcumin was recently discovered to have wound healing aspects, a property which stems from its antioxidant effect. Curcumin of the o-methoxy phenol derivative plays a role in enzyme detoxification, reducing oxidative stress through reactive oxygen species reaction. This process, in turn, then lessens oxidation and inflammation at the wound site when applied topically through oxidative damage inhibition [129, 130]. Curcumin-based scaffolding promotes granulation tissue development, which aids re-epithelialisation by providing structural integrity for epithelial cells to migrate and repair the wound gap [131].

Curcumin can accelerate the wound healing phases from the inflammatory phase to proliferative phase by increasing neovascularisation, collagen deposition, advanced re-epithelialisation and tissue formation [132]. Curcumin applied to a skin wound has been found to increase TGF-$\upbeta$ as well as an increase in synthesis of ECM, both of which aid in wound contraction [133]. The compound also aids in accelerating wound healing by preventing DNA break down and lipid peroxidation [134]. Curcumin can be administered orally and topically, to which the latter is favoured in wound healing for higher bioavailability at the site of injury [130]. However, curcumin also possesses several disadvantages in therapeutic use such as poor solubility in liquids, compound instability, short half-life in plasma, inefficient tissue absorption, and light-sensitive [129, 135]. To overcome these disadvantages, reducing curcumin to the nano scale may aid in improving its properties.

A study conducted by Li et al. demonstrated the use of curcumin nanoparticles (CNPs) incorporated into methoxypoly(ethylene glycol)-graft-composite film and applied onto Sprague-Dawley rats’ excision wounds. The CNPs were theorised to be able to overcome the solubility and stability issues that the common curcumin drug poses. After 2 weeks, the composite film showed improved wound healing with roughly 90% wound reduction compared to a 60% wound reduction in the composite film without CNPs. Besides that, the researchers have also studied the incorporation of CNPs into N,O-carboxymethyl chitosan/oxidised alginate hydrogel on full-thickness mice excision wounds. The hydrogel composite displayed faster wound healing, in which after 2 weeks the wound size was exceptionally reduced with edging full wound closure [136].

Besides that, one other study incorporated curcumin into PLGA nanoparticles through emulsification-solvent evaporation process. The nanoparticles successfully delivered curcumin in a sustained fashion intradermally and increased wound healing speed in a mouse model. Histological studies demonstrated wound treated with PLGA-curcumin nanoparticles had increased collagen concentration, granulation tissue, and wound maturation in the remodelling phase [137].

### Dendrimers

**Fig. 4:**
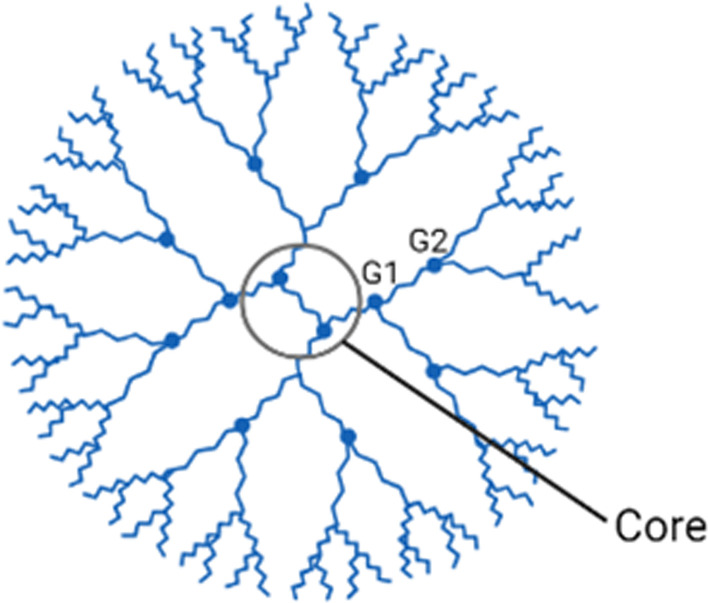
Dendrimer structure. Created with BioRender.com

Dendrimers are organic nanoparticle polymers typically characterised by their unique symmetrical branching structures stemming from a core (Fig. 4). Dendrimers are globular, homogenous, mono dispersed with radial symmetry, containing various anionic, neutral, or cationic surface charge properties [138, 139]. Dendrimers have low immunogenicity, low cytotoxicity, as well as hepatic and renal secretory pathways [140]. From these characteristics, dendrimers are suitable candidates for drug and gene delivery systems [141, 142]. Various therapeutic agents have solubility, stability, or toxicity issues when used directly on the human body, thus the use of dendrimers as a carrier can alleviate these problems and better deliver the compounds [141]. Dendrimers can also be customised by branch length, dendritic scaffold target synthesis, size, shape, surface functionality, and more to aid in drug delivery [143]. Dendrimers have been utilised to aid in the treatment of inflammatory diseases, cancer, and cardiovascular diseases [144–146]. However, some studies displayed that dendrimers alone can be of medicinal use, as it possesses antibacterial and antifungal properties [147]. It is suggested that the dendrimer terminal amine groups affect the integrity of bacterial membranes, effectively exerting antibacterial activity. Dendrimer functional groups adhere to the bacterial cell surfaces and diffuse through the cell wall. By binding to the cytoplasmic membrane, the dendrimer functional groups cause nucleic material release like DNA and RNA as well as electrolytes from the gradual cytoplasmic membrane break down [148]. These characteristics enforce that dendrimers are a suitable candidate for wound healing usage.

One study by Jiang et al. utilised mannose-decorated globular lysine dendrimers (MGLDs) in its effect on diabetic wound healing. The researchers found that MGLDs had excellent biocompatibility, and induced cell surface mannose receptor clustering on mouse bone marrow-derived macrophages, which then increased various growth factor and interleukin production as well as fibroblast proliferation. MGLDs also increased the rate of full-thickness wound healing through M2 macrophage polarisation in type 2 diabetic mice. It improved wound closure rates, collagen deposition, and angiogenesis to the damaged site during the proliferative stage [149].

Another study by M. Vedhanayagam and colleagues incorporated zinc oxide (ZnO) nanoparticles into triethoxysilane poly(amidoamine) dendrimer generation 1 (TES-PAMAM-G1) on collagen scaffold to be utilised in wound healing. The nanostructure demonstrated better thermal and mechanical stability than the normal collagen scaffold. In vivo wound healing tests with the scaffold demonstrated superior re-epithelialisation and collagen deposition, likely due to the contribution of ZnO nanoparticles improving end-stage inflammation, angiogenesis, and collagen matrix formation, resulting in accelerated wound healing [150]. The ZnO dendrimer nanoparticles aided in reducing inflammatory cytokines, chemokines, and secretory cell adhesion molecules, which are all biomarkers for inflammation [151].

Based on the organic biomaterials mentioned, several comparative and/or collaborative analyses have been done to assess the effectiveness of these biomaterials. Tripathi et al. conducted a comparative analysis between collagen and chitosan as wound dressings and its haemostatic effect. Chitosan gauze demonstrated better binding affinity of erythrocytes, as well as a shorter haemostasis time compared to collagen gauze [152]. While this study was only conducted on regular collagen and chitosan but not in nano-size, this information may be a useful reference to future studies of the biomaterials in nanoparticle form. One study developed chitosan-collagen scaffolds in nano/microfibrous structure and was found to encourage keratinocyte migration and wound re-epithelialisation tested on ex vivo human skin [153]. Another study fabricated collagen and chitosan scaffolds loaded with curcumin nanoparticles and was found to have accelerated wound healing progression through the increase of epidermal thickness, angiogenesis upregulation, and upregulated collagen deposition to the wound site as compared to the control group [154].

Another study loaded antileishmanial agents amphotericin B and betulinic acid each into chitosan and dendrimer nanoparticles and found that while both loaded nanoparticles successfully eliminated *L. major* infection, chitosan nanoparticles loaded with both the antileishmanial agents individually were more effective than its dendrimer counterparts [154]. While dendrimers are useful biomaterials for wound healing, it can be accompanied by certain drawbacks. Yallapu et al. found dendrimer curcumin nanoformulations demonstrated toxicity towards red blood cells compared to other forms of curcumin nanoformulations by reducing the biconcavity of the blood cells, which can affect the bleeding and haemostasis phase of wound healing [155, 156].

### Graphene

Graphene is a substance produced from graphite that is composed of pure carbon, one of nature’s most significant elements found in everyday products such as pencil lead. Graphene is distinguished by its toughness, flexibility, lightness, and excellent resistance [157]. Graphene oxide (GO) is a category in which graphene-based nanomaterials are classified. Due in large part to its adaptable physicochemical features, excellent biocompatibility, and simple accessibility, GO is a topic of great interest and potential in the biomedical sciences [158].

#### Graphene oxide

One monomolecular layer of graphite with multiple oxygen-containing functions, including epoxide, carbonyl, carboxyl, and hydroxyl groups, makes up graphene oxide (GO). GO can be fabricated from reducing graphite to GO via chemical syntheses such as Brodie’s, Hummers, or Offeman methods. In addition to chemical synthesis, electrochemical synthesis is used to create GO. This form of synthesis produces safe GO with a regulated oxidation state. It is interesting to note that sugarcane bagasse has been utilised in the synthesis of GO. Similarly, oxidising bacteria like *Acidithiobacillus ferrooxidans* or *Pseudomonas* have also been utilised to transform graphite into GO [159]. Due to the presence of greater oxygen-containing groups than rGO, GO has high wettability and water solubility. Since GO is easily functionalised and changed, it offers enormous potential for enhanced drug delivery [158].

Notably, broad-spectrum efficacy against bacterial pathogens is displayed by carbon allotropes. *Proteus mirabilis*, *Salmonella typhi*, *Klebsiella pneumoniae*, and *Serratia marcescens* are pathogens that are effectively combated by the potent bactericidal activity of GO [160]. GO causes cell damage, which is followed by reactive oxygen species production and cell death. According to Tu et al., significant dissipation reactions among graphene and lipid molecules allow graphene nanosheets to remove major quantities of phospholipids from E.coli cell membranes, effectively presenting bactericidal activity [161]. On the surface of bacteria, disulphide linkages are always present in order to preserve a specific protein shape. The protein structure is connected to the configuration of these bonds. Based on the Raman spectroscopy assessment, when the concentration of GO in the *E. coli* and *E. faecalis* culture was increased, the vibrational intensity of the adenine and protein bands rose, subsequently destabilising it and result in the death of these bacteria [162].

The design of GO with sp2 hybridised carbon has a high surface area, allowing high concentrations of pharmaceuticals to be deposited on both sides of a single atomic layer sheet, making it an appropriate agent for drug administration [163]. A GO-cellulose nanocomposite was created by Soliman et al. and its wound healing effectiveness was assessed. In vitro wound scratch assay demonstrated the biocompatibility and cell migration of the GO nanocomposite. The GO nanocomposite enhanced wound closure by 80% on the 20th day, according to the in vivo studies. On the 21st day, the GO nanocomposite induced angiogenesis, re-epithelialisation, and an increase in collagen synthesis, successfully progressing the wound healing proliferative phase [164].

Electrospun PCL was coated with GO as an implantable patch was fabricated and loaded with high concentrations of ibuprofen (5.85 mg cm^-2^), ketoprofen (0.86 mg cm^-2^), and vancomycin (0.95 mg cm^-2^), which were used as anti-inflammatory and antibiotic models. In this study, an electrospun PCL patch that has been functionalised with GO by nitrogen plasma activation is developed for the on-demand diffusion of nonsteroidal anti-inflammatory medicines and antibiotics to injury sites in response to near infrared light stimulation. At the biomaterial composite surface, near infrared light was acquired by GO sheets, which then allowed local heating and drug release through vibrational motion. Near infrared light phototherapy stimulation enables the implantable patch to respond efficiently and release the payload on demand for longer than three days. This indicates that GO functions effectively as a drug delivery system as well as responds positively to phototherapy [165].

## Nanoparticle drug delivery systems

**Fig. 5:**
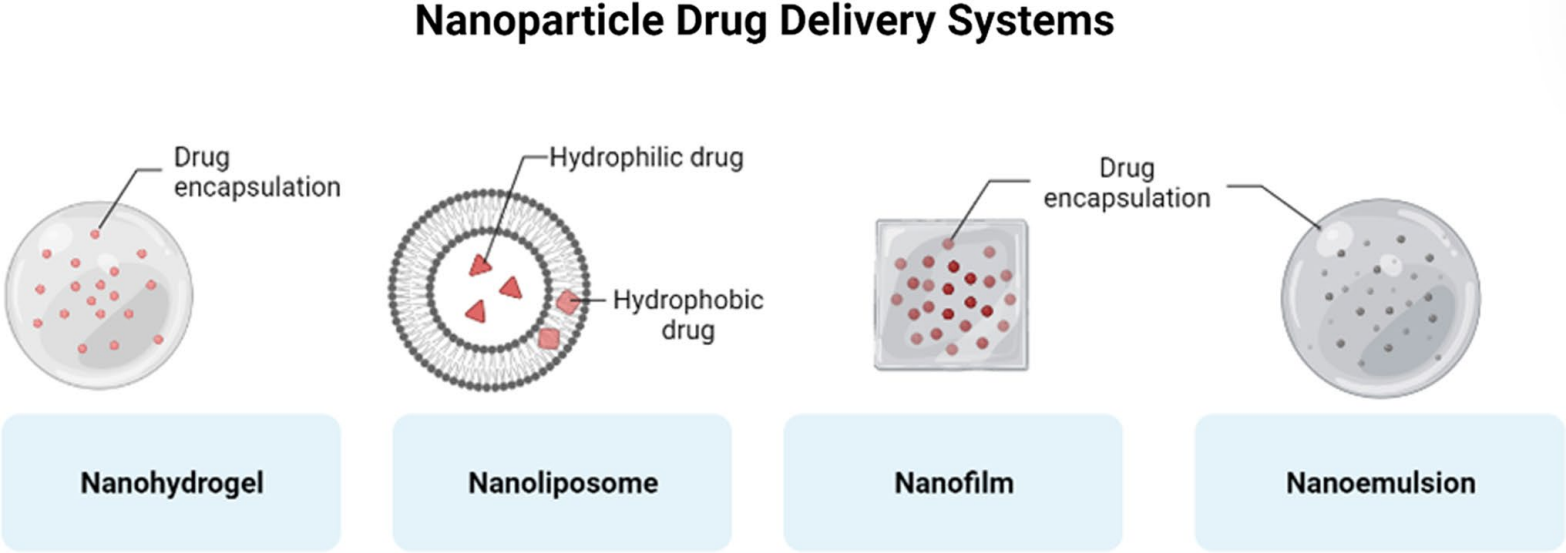
Nanoparticle drug delivery systems. Adapted from “Nanoscale Delivery Approaches for Small-Molecule Cargoes”, by BioRender.com (2023). Retrieved from https://app.biorender.com/biorender-templates

An alternative to delivering therapeutic agents to wound sites is to incorporate it into nanoparticles drug delivery systems such as nanohydrogels, nanoliposomes, nanofilms, and nanoemulsion (Fig. 5) [49]. In vivo destabilisation, limited bioavailability and dissolution, low body integration, issues with target-specific dissemination and efficacy, and possibly hazardous pharmacological consequences are major challenges when using large-scale materials for drug delivery. Consequently, utilising novel drug delivery techniques to target treatments to specific areas of the human physique may be a potential solution to address these urgent issues. Therefore, regulated medication release, present drug formulations, and effective drug delivery may all be significantly impacted by nanotechnology [166]. In many instances, nanocarriers are just as important as therapeutic nanomaterials itself to successfully deliver molecules/compounds to wound sites. Nanosized drug delivery systems are favoured and highly researched due to its capability in increasing drug delivery efficiency and efficacy, as well as delivering therapeutic agents in a controlled manner and increase chemical activity [167].

### Nanohydrogels

Nanohydrogels are three-dimensional nanostructure clusters that presents high mechanical strength, wound exudate absorption and moisture retention, as well as high hydrophobic and hydrophilic drug delivery efficacy due to their porous physicality [49, 168, 169]. Large three-dimensional structures of hydrogels, which may cause the active therapeutic ingredient to diffuse through its matrix in a swelled state, are significant limitations to its application. Hydrogel nanoparticles or nanohydrogels can easily address these shortcomings. Hydrogel and nanoparticle drug delivery are both qualities of nanogels. As a result, they benefit from a variety of hydrogel characteristics, including their hydrophilic nature, flexibility, adaptability, high water absorption, and biocompatibility, as well as the numerous benefits attributed to nanoparticulate systems. With a regulated release in the target area for days or even weeks, nanohydrogels demonstrate superior therapeutic efficacy at the site of action via simple diffusion into tissues [170].

One experiment by Montanari et al. demonstrated the self-assembled hyaluronan-based nanohydrogels use in encapsulating natural antioxidants like astaxanthin, resveratrol, and curcumin. The study has shown the nanohydrogel to increase water solubility of the encased natural antioxidants in aqueous media, display ideal hydrodynamic diameters as well as effective reactive oxygen species neutralisation [168]. Li et al. fabricated micro-/nanoscaled hydrogels from alginate-gum arabic which contains binding properties to damaged tissues, enhance cellular proliferation and differentiation, together with controlling bleeding [171].

### Nanoliposomes

Nanoliposomes are spherical colloidal vessels made of a phospholipid bilayer and an aqueous center within the nanometer scale, which has been found to increase bioavailability, as well as enhance the controlled release and solubility of therapeutic agents [172]. Nanoliposomes are regarded as improved drug delivery vehicles because the membrane structure displays similar characteristics to that of cell membranes, which allow for effective pharmaceutical incorporation [173]. Polymeric nanoliposome fabrication for wound healing was discovered to improve dermal penetration in the delivery of therapeutic agents and increase product retention duration [49]. Kotwal et al. fabricated unilamellar lipid nanovesicles encasing adenosine triphosphate (ATP), to which the nanoliposomes fuse with cell membranes and release ATP to the cytoplasm. This nanoliposome has reduced the wound healing time by increasing macrophages to wound site, encouraging neovascularisation and collagen production from VEGF influx [174].

### Nanofilms

Nanofilms are layering films in the nanometer size, which are primarily used for layering cells in medical research. The nanofilms are often made with therapeutic agents, utilising a layer-by-layer assembly method with cells via molecular interactions to fabricate a biocompatible system. Its fabrication can be customised with various molecules such as antibodies, proteins and growth factors depending on the targeted ailment, stabilised and consolidated to prevent reduced efficacy as well as disruption within aqueous solutions [175]. Nanofilms can be used to overcome the shortcomings of biomaterials with single layer fabrication such as hydrogels, which present disadvantages like rapid release of the integrated therapeutic agents and low mechanical stability [176]. One study conducted by Matsusaki et al. utilised fibronectin and gelatin, both of which are easily sourced form nature, to fabricate nanofilms. These nanofilms then act as a nano-ECM in between layers of cells [177]. Naturally sourced ECM components in this biomaterial are important in preventing cytotoxicity. The author selected fibronectin due to it being a glycoprotein involved in cellular adhesion, migration, and differentiation whilst having a collagen binding domain, giving it the ability to bind to gelatin [178]. The nanofilm layering system successfully formed vascularised tissue, making it a promising development in in vitro 3D tissue modelling [177].

### Nanoemulsions

Nanoemulsions are solid sphere colloidal particles that can act as a therapeutic agent carrier in the nanometer range [179]. Nanoemulsions aid in enhancing the bioavailability of drugs from its encapsulation, dissolve hydrophobic therapeutic agents, and prevent drug degradation while maintaining long-term stability [180]. While nanoemulsion fabrication typically focuses on drug delivery, other natural therapeutic alternatives such as plants essential oils can also be encapsulated [49]. Plant essential oils have considerable medical research due to the presence of high bioactive compounds [181]. Alam et al. encapsulated clove oil into nanoemulsion, to which the study determined that it is biocompatible and non-toxic whereby the nanoemulsion induced notable wound healing effects and increased leucine content with no immune cells activated [182]. Another study utilised nanoemulsion constituting riboflavin-stabilised gelatin and carvacrol from oregano oil in preventing skin wound bacterial infections. This nanoemulsion demonstrated broad-spectrum antimicrobial activity on drug-resistant bacterial biofilms as well as significantly reduced wound healing time in vivo [183].

## Limitations of existing research

In summary, it has been shown that nanomaterials are beneficial for various stages of the wound healing process. However, a high level of uncertainty regarding the potential risks and real benefits of nanoparticles and nanomedicines presented notable barriers along its translational development. Human trials of nanotechnology-based medicinal applications are particularly interesting in this regard since it raises the highest level of unpredictability in all clinical areas. Although research on nanomaterials used in medicine has increased over time, it has largely been directed toward technological advancement rather than the identification of potential risks associated with nanoproducts. As a result, nanosafety has continued to receive insufficient consideration [184].

When certain nanoparticles are mixed with water, air, or biological medium, it may agglomerate to become larger sized particles. Therefore, it is crucial to conduct a thorough and extensive morphological, physico-chemical, and biological characterisation of nanomaterials that have been produced. By utilising techniques already used and validated in nanotoxicology, as well as improving the physico-chemical characterisation, we can investigate the potential cytotoxic effects caused by the degradation and release of nanomaterials in the biological environment, as well as the functionalisation with organic compounds. The high cost of nanomaterial formulations could also prevent its practical applications [185].

## Conclusion

In conclusion, there are a myriad of organic and inorganic nanomaterials which can be used for wound healing purposes. While it is still common to obtain nanomaterials from finite sources or with environmentally damaging fabrication methods such as electrospinning, arc discharge, laser ablation, nanoimprint lithography, chemical vapor deposition, atomic layer deposition, electrodeposition, and molecular beam epitaxy, new alternate biological methods are increasingly being used, sourcing nanoparticles from plants and microorganisms alike. Reducing various organic and inorganic materials to nano sizes can also bring about increased benefits to wound healing compared to its regular sized counterparts. In inorganic nanoparticles, metal nanoparticles like silver, titanium dioxide, copper oxide, and cerium oxide were discussed in detail on its antibacterial effects. Other inorganic nanoparticles like MXenes, PLGA, PEG, and silica nanoparticles were also mentioned. On the other hand, organic nanoparticles like collagen, chitosan, curcumin, dendrimers, graphene and its derivative graphene oxide were detailed on its respective contributions to wound healing. Nanoparticle delivery systems also utilise nanohydrogels, nanoliposomes, nanofilms, and nanoemulsions to act as a carrier for drugs and/or therapeutic agents in wound healing. Due to the various limitations present in nanomaterials, more research is required to further perfect nanomaterial fabrication and safety in hopes that soon, these biomaterials will be more sustainable and accessible for people struggling with wound healing.

## Data Availability

Not applicable.

## Abbreviations

PECAM-1: Platelet endothelial cell adhesion molecule 1
TGF-$\upbeta$: Transforming growth factor-beta
EGF: Epidermal growth factor
ECM: Extracellular matrices
DMOG: Dimethyloxalylglycine
PCL: Poly($\upepsilon$-caprolactone)
PCLF/DMOG: Dimethyloxalylglycine-embedded poly($\upepsilon$-caprolactone) fiber
PVA: Polyvinyl alcohol
VEGF: Vascular endothelial growth factor
PDGF: Platelet-derived growth factor
FGF: Fibroblast growth factor
MMPs: Mixed metalloproteinases
EMT: Epithelial-mesenchymal transition
bFGF: Basic fibroblast growth factor
PLGA: Poly(lactic-co-glycolic acid)
AgNP: Silver nanoparticle
TiNP: Titanium dioxide nanoparticle
CuNP: Copper oxide nanoparticle
HIF: Hypoxia-inducible factor
CeNP: Cerium oxide nanoparticle
2D: Two-dimensional
PGE: Poly(glycerol-ethylenimine)
T-RMFs: MXene nanobelt fibers
PAAV: P(AAm-co-AN-CO-VIm)
AMX: Antibiotic amoxicillin
SiNP: Silica nanoparticle
CNP: Curcumin nanoparticle
ZnO: Zinc oxide
TES-PAMAM-G1: Triethoxysilane poly(amidoamine) dendrimer generation 1
ATP: Adenosine triphosphate
GO: Graphene oxide
MRSA: Methicillin-resistant *Staphylococcus aureus*
